# The mediating role of depression on the association between physical activity and cognitive function among older adults

**DOI:** 10.3389/fnagi.2025.1470256

**Published:** 2025-09-05

**Authors:** HuanRui Zhang, Wen Tian, GuoXian Qi, XiuFang Wei

**Affiliations:** Department of Geriatric, The First Hospital of China Medical University, Shenyang, China

**Keywords:** physical activity, depression, cognitive function, older adult, mediation analysis, NHANES

## Abstract

**Background:**

Previous studies had showed that physical activity (PA) can effectively reduce cognitive decline. Nonetheless, it is still unclear whether depression can mediate the relationship between PA and cognitive decline.

**Methods:**

This study encompassed 2,681 older adults (≥ 60 years) from the National Health and Nutrition Examination Survey (NHANES) study during 2011–2012 and 2013–2014 cycles. PA was assessed, including recreation activity, work activity, and walking/bicycling. Depression was evaluated using the Patient Health Questionnaire-9, and cognitive function was assessed through a series of cognitive tests at the Mobile Examination Center. Utilizing weighted multivariable linear regression, we assessed the associations among PA, depression, and cognitive function. Additionally, a mediation model was employed to investigate how depression mediates the relationship between PA and cognitive decline.

**Results:**

We found that only moderate to high-intensity recreation activity and depression were associated with better cognitive function, including performance on the Animal Fluency Test (AFT), the Digit Symbol Substitution Test (DSST), and the overall cognitive function (composite z-score), following adjustments for potential confounding factors. Depression emerged as a mediator in the relationship of moderate to high-intensity recreation activity with AFT, DSST, and the composite z-score, mediating 6.5%, 12.3%, and 10.5% of the overall association, respectively. Furthermore, in the sensitivity analysis that excluded participants with a history of stroke, the sensitivity analysis results remained consistent and stable.

**Conclusion:**

This study found that in older adults, increasing engagement in moderate to high-intensity recreation activity, rather than work activity or walking/bicycling, is related with a reduction in cognitive decline. Notably, depression emerged as a pivotal mediating factor in this relationship.

## Introduction

The global aging population is growing rapidly, with individuals aged 65 and older accounting for approximately 9% in 2019 and projected to reach 16% by 2050 (Huang and Ren, 2022). With this demographic shift, age-related cognitive decline has emerged as a significant public health challenge (GBD 2017 US Neurological Disorders Collaborators, Feigin et al., 2021). Various factors contribute to cognitive decline, such as neurodegeneration caused by extracellular accumulation of amyloid plaques and hyperphosphorylation of tau protein, cerebrovascular disease, infection, trauma, and nutritional metabolic disorders (Apostolo et al., 2016; Lane et al., 2018). If prevention and intervention measures are not implemented, cognitive disorders may progress to mild cognitive pathological impairment (MCI) and potentially even dementia, for which effective treatments remain limited (Wang et al., 2021; Chen et al., 2022). Therefore, there is a critical need to identify modifiable factors influencing cognitive function and potential underlying mechanisms.

One potentially valuable intervention is physical activity (PA). Consistent evidence shows that PA offers protective benefits against numerous chronic diseases (Bull et al., 2020), and several studies have indicated its protective role against cognitive decline (Obisesan et al., 2012; Loprinzi et al., 2018; Zheng et al., 2022). A systematic study exploring the association between PA and cognitive function among individuals aged 60 and older has found the positive impact of PA on cognitive function (Carvalho et al., 2014). Older adults could benefit from better regulation of hippocampal function, neurogenesis, brain blood flow, as well as a reduction in proinflammatory activity following PA (Carvalho et al., 2014; Silva et al., 2019; Chu et al., 2021). Furthermore, dose-response studies have demonstrated that higher levels of physical activity are associated with better cognitive function in older adults (Carvalho et al., 2014; Loprinzi et al., 2018; Zheng et al., 2022). However, the relationships between various types of PA (recreation activity, work activity, and walking/bicycling) and cognitive function remain insufficiently understood.

Recent studies have indicated that PA can serve as both a therapeutic and preventive strategy for depression (Kandola et al., 2019). A meta-analysis of prospective cohort studies showed that PA provides protection against the onset of depression, regardless of age and geographical region (Schuch et al., 2018). Among older adults, individuals who engage in higher levels of PA exhibit a reduced risk of developing depression both currently and in the future (Marques et al., 2020). Accumulating evidence suggests a significant association between depressive symptoms and impaired cognitive functions (Snyder, 2013; Demakakos et al., 2017). This may be attributed to the fact that depression and cognitive decline share certain common pathological mechanisms. Several studies have proposed that depression may represent an early clinical manifestation during the preclinical stage of dementia syndrome (Gatchel et al., 2019; Wu et al., 2024). Current research has demonstrated that late-life depression is often associated with a more rapid decline in cognitive abilities (Jung et al., 2022). Notably, the association between depressive symptoms and poorer cognitive performance has been found to be strongest during later stages of life (Donovan et al., 2017; Wei et al., 2019; Jung et al., 2022). Therefore, we hypothesize that depression plays a crucial role in the pathway linking PA to cognitive function. This study utilizes data from the National Health and Nutrition Examination Survey (NHANES) to investigate the moderating role of depression in the association between PA and cognitive function.

## Materials and methods

### Study design and patients

**FIGURE 1:**
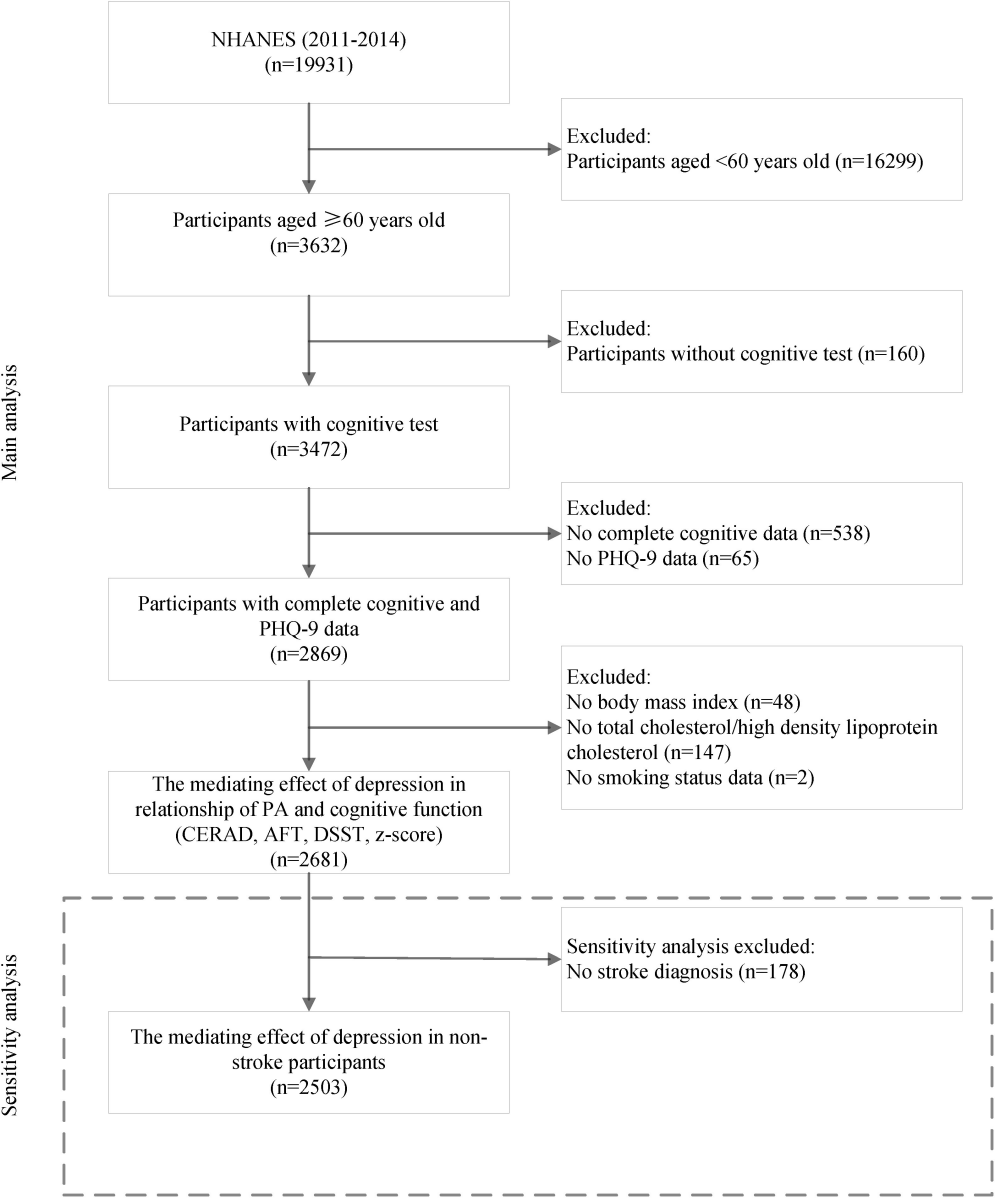
Flow chart of participants selection.

NHANES is a biennial, population-based survey with a multi-level probability sampling design, aimed at assessing the health status of representative United Stated residents. For this study, we used data from the 2011–2012 to 2013–2014 NHANES cycles, which are two independent cross-sectional surveys. These cycles were selected because cognitive function assessments were included during these years. A total of 2,869 older adults (≥ 60 years) with complete cognitive test results and depression assessments were recruited. After further excluding those with incomplete confounding factors (*N* = 188), 2,681 participants were retained for final analysis (Figure 1). The ethics review committee of the National Center for Health Statistics approved protocols for the NHANES study, and subjects signed the informed consent form. In this study, the local hospital ethics committee’s approval was waived because data were publicly accessible and de-identified.

### Physical activity assessment

All information regarding PA in this study was self-reported and obtained through the Physical Activity Questionnaire in NHANES 2011–2014. The criteria recommended by the WHO to achieve satisfactory levels of PA include any one of the following items (Bull et al., 2020): (1) engaging in moderate intensity aerobic PA for at least 150 min per week; (2) engaging in high-intensity aerobic PA for at least 75 min per week; or (3) an equivalent combination of moderate and high-intensity aerobic PA (with 1 min of vigorous-intensity PA considered equivalent to 2 min of moderate intensity PA), totaling at least 150 min per week.

In terms of work activity, older adults were divided into insufficient activity (< 150 min/week) and sufficient activity (≥ 150 min/week) of moderate to high-intensity work activity. Similarly, for recreation activity, older adults were categorized based on insufficient activity (< 150 min/week) and sufficient activity (≥ 150 min/week) of moderate to high-intensity recreation activity. Regarding walking/bicycling activities, older adults were classified as having insufficient activity (< 150 min/week) and sufficient activity (≥ 150 min/week) for walking/bicycling.

### Depression assessment

Depression was examined using the Patient Health Questionnaire-9 (PHQ-9) (Kroenke et al., 2001). Each item of PHQ-9 is scored from 0 to 3, with total score ranging from 0 to 27; higher scores indicate greater severity of depression. We consider the total score as the mediating factor in the study.

### Cognitive function assessment

During NHANES 2011–2012 and 2013–2014 cycles, cognitive function in older adults was evaluated through a series of cognitive assessments in the Mobile Examination Center (MEC). Two subtests from the Consortium to Establish a Registry for Alzheimer’s Disease (CERAD)-immediate recall and delayed recall-were administered, along with the Animal Fluency Test (AFT) and the Digit Symbol Substitution Test (DSST). Detailed descriptions of these cognitive tests were provided in our previous publication (Zhang et al., 2022). The scores from the three cognitive tests were averaged to calculate the composite score (z-score). The CREAD total score, AFT total score, DSST total score, and composite z-score were used as cognitive function outcomes in this study.

### Covariates

Population demographics comprise age, sex, race (non-Hispanic White, non-Hispanic Black, Hispanic, and other), education level (< 11th grade, ≥ 11th grade), and smoking habits (never, former, and current). Data on comorbidities were also collected, including levels of blood lipids (ratio of total cholesterol to high density lipoprotein cholesterol), hypertension, diabetes, and cardiovascular disease (CVD). Medication history encompass lipid-lowering, antihypertensive, hypoglycemic, antiplatelet drugs. A diagnosis of heart failure, coronary heart disease, angina, myocardial infarction, or stroke by a physician was used to define cardiovascular disease.

### Statistical analysis

Statistical analyses were conducted using in R software (version 4.0.3), with significance set at *P* < 0.05. We accounted for the complex, multistage probability sampling design of NHANES. To ensure nationally representative estimates and valid statistical inferences, we applied the appropriate sample weights (WTMEC2YR × 1/2) provided by NHANES following the manual of NHANES. Weighted means with standard errors (SE) were employed to describe the continuous variables, while weighted proportions were applied for categorical variables. Utilizing weighted multivariable linear regression to estimate the coefficients and 95% confidence intervals (CI) of the associations among PA, depression, and cognitive function. In the crude model, no variables were adjusted, while in the adjusted model, adjustments were made for age, sex, race, marital status, education level, BMI, smoking habits, ratio of total cholesterol to high density lipoprotein cholesterol, hypertension, diabetes, CVD, and mediation use (lipid-lowering, antihypertensive, hypoglycemic, and antiplatelet drugs). Furthermore, the mediating effect of depression in the relationship between PA and cognitive function was examined using the “mediation” package-based mediation model. To ensure the robustness of the study, a mediation analysis was further conducted in the non-stroke population.

## Results

**FIGURE 2:**
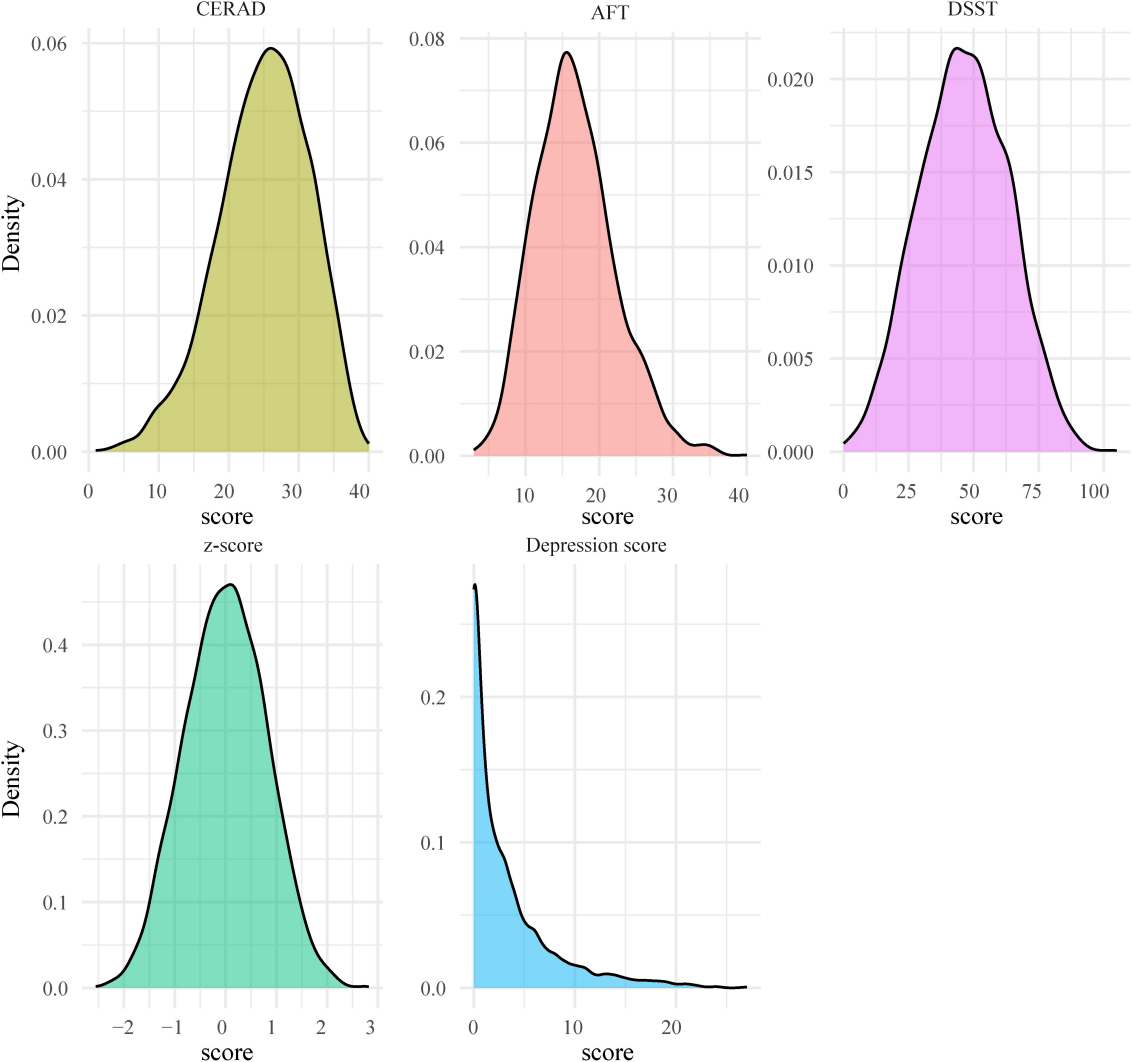
Density plots showed the distributions of depression and cognitive scores among older adults. The plots illustrate the distribution of depression scores (measured by PHQ-9), along with Consortium to Establish a Registry for Alzheimer’s Disease (CERAD), Animal Fluency Test (AFT), Digit Symbol Substitution Test (DSST), and composite cognitive z-scores.

Table 1 presented the basic characteristics of 2,681 older adults, representing a weighted sample of 49,539,645 participants. The weighted mean age was 69.13 years, and the weighted proportion of males was 45.94%. Meanwhile, the majority ethnicity of older adults belonged to non-Hispanic White (80.25%), followed by other races (8.22%), non-Hispanic Black (7.89%), and Mexican American/Hispanic (3.64%). Moreover, there were approximately 84.4% participants undergoing education > 12 years and 62.34% participants were married or living with a partner. In terms of moderate to high-intensity recreation activity, moderate to high-intensity work activity, and walking/bicycling, the weighted proportion with ≥ 150 min/week was 30.21%, 25.15%, and 12.04%, respectively. In addition, the weighted mean scores of depression symptoms, CERAD, AFT, and DSST were 2.83, 26.06, 18.16, and 52.52, respectively, and the distribution of the data was presented in the form of kernel density plots in Figure 2.

**TABLE 1:** Descriptive statistics of all participants.

Variables	All participants (*n* = 2,681)
Weighted number	49,539,645
Age, years, mean ± SE	69.13 ± 0.19
Sex-male, % (SE)	45.94 (0.98)
Ethnicity, % (SE)	
Non-Hispanic White	80.25 (1.82)
Non-Hispanic Black	7.89 (1.09)
Mexican American/Hispanic	3.64 (0.66)
Other	8.22 (0.91)
Education > 12 years, % (SE)	84.4 (1.45)
Marital-married/with a partner, % (SE)	62.34 (1.2)
Body mass index, kg/m^2^, mean ± SE	29.04 ± 0.22
Smoking, % (SE)	
Never	50.08 (1.49)
Former	38.75 (1.25)
Current	11.17 (0.77)
Total cholesterol/high density lipoprotein cholesterol, mean ± SE	3.71 ± 0.04
Hypertension, % (SE)	66.64 (1.23)
Diabetes, % (SE)	23.88 (1.01)
Cardiovascular disease, % (SE)	21.35 (0.99)
Lipid-lowering drugs,% (SE)	43.67 (1.46)
Antihypertensive drugs,% (SE)	51.79 (1.65)
Hypoglycemic agents, % (SE)	17.57 (1.01)
Antiplatelet drugs, % (SE)	5.97 (0.85)
Moderate to high-intensity recreation activity ≥ 150 min/week, % (SE)	30.21 (1.46)
Moderate to high-intensity work activity ≥ 150 min/week, % (SE)	25.15 (1.71)
Walking/bicycling ≥ 150 min/week, % (SE)	12.04 (0.92)
Depression score, mean ± SE	2.83 ± 0.12
CERAD: total Score, mean ± SE	26.06 ± 0.32
AFT: total Score, mean ± SE	18.16 ± 0.18
DSST: total Score, mean ± SE	52.52 ± 0.56

Continuous variables are presented as weighted means and standard errors, while categorical variables are presented as weighted percentages with standard errors. CERAD, Consortium to Establish a Registry for Alzheimer’s disease Word Learning sub-test; AFT, Animal Fluency Test; DSST, Digit Symbol Substitution Test.

Employing weighted multivariable linear regression, we estimated the associations between PA, depression, and cognitive function in both crude and adjusted model (Table 2). After adjusting for confounders, the depression score was negatively associated with AFT (β:−0.110; 95% CI: −0.179 to −0.041), DSST (β: −0.513; 95% CI: −0.764 to −0.262), and z-score (β:−0.021; 95% CI: −0.033 to −0.010), but not associated with CERAD. In addition, in adjusted model, sufficient moderate to high-intensity recreation activity was positively correlated with AFT (β: 1.764; 95% CI: 1.206 to 2.321), DSST (β: 3.382; 95% CI: 1.709 to 5.054), and z-score (β: 0.199; 95% CI: 0.121 to 0.276), but not correlated with CERAD. However, in fully adjusted model, there was no significant association between sufficient moderate to high-intensity work activity and walking/bicycling with cognitive function.

**TABLE 2:** Associations between physical activity and depression score with cognitive function [β (95% CI)].

Depression score/physical activity	Weighted number (%)	CERAD: total score	*P*-value	AFT: total score	*P*-value	DSST: total score	*P*-value	Z-score	*P*-value
**Crude model^£^**									
Depression score	49,539,645 (100)	−0.103 (−0.198, −0.009)	0.033	−0.177 (−0.242, −0.111)	< 0.001	−0.752 (−1.018, −0.485)	< 0.001	−0.031 (−0.043, −0.018)	< 0.001
**Moderate to high-intensity recreation activity (min/week)**									
< 150	34,575,950 (69.8)	Ref		Ref		Ref		Ref	
≥ 150	14,963,694 (30.2)	1.145 (0.438, 1.853)	0.002	2.574 (1.934, 3.213)	< 0.001	7.019 (5.002, 9.036)	< 0.001	0.353 (0.262, 0.443)	< 0.001
**Moderate to high-intensity work activity (min/week)**									
< 150	37,079,450 (74.8)	Ref		Ref		Ref		Ref	
≥ 150	12,460,195 (25.2)	0.729 (−0.023, 1.481)	0.057	0.877 (0.165, 1.589)	0.017	3.382 (0.759, 6.004)	0.013	0.157 (0.040, 0.274)	0.010
**Walking/bicycling (min/week)**									
< 150	43,574,216 (88.0)	Ref		Ref		Ref		Ref	
≥ 150	5,965,429 (12.0)	0.431 (−0.540, 1.401)	0.372	1.117 (−0.015, 2.249)	0.053	0.426 (−1.927, 2.779)	0.714	0.099 (−0.028, 0.226)	0.123
**Adjusted model^§^**									
Depression score	49,539,645 (100)	−0.087 (−0.183, 0.008)	0.071	−0.110 (−0.179, −0.041)	0.004	−0.513 (−0.764, −0.262)	0.001	−0.021 (−0.033, −0.01)	0.002
**Moderate to high-intensity recreation activity (min/week)**									
< 150	34,575,950 (69.8)	Ref		Ref		Ref		Ref	
≥ 150	14,963,694 (30.2)	0.490 (−0.230, 1.210)	0.165	1.764 (1.206, 2.321)	< 0.001	3.382 (1.709, 5.054)	0.001	0.199 (0.121, 0.276)	< 0.001
**Moderate to high-intensity work activity (min/week)**									
< 150	37,079,450 (74.8)	Ref		Ref		Ref		Ref	
≥ 150	12,460,195 (25.2)	0.024 (−0.638, 0.685)	0.940	−0.108 (−0.732, 0.515)	0.714	0.139 (−2.020, 2.298)	0.892	−0.003 (−0.097, 0.091)	0.951
**Walking/bicycling (min/week)**									
< 150	43,574,216 (88.0)	Ref		Ref		Ref		Ref	
≥ 150	5,965,429 (12.0)	0.079 (−0.879, 1.036)	0.862	0.566 (−0.696, 1.828)	0.350	−1.103 (−3.254, 1.048)	0.288	0.017 (−0.111, 0.145)	0.777

^£^ Crude model adjusted for no confounding variables.

^§^ Adjusted model adjusted for age, gender, ethnicity, marital, education, BMI, smoking, total cholesterol/high density lipoprotein cholesterol, hypertension, diabetes, cardiovascular disease, lipid-lowering drugs, antihypertensive drugs, hypoglycemic agents, antiplatelet drugs. CERAD, Consortium to Establish a Registry for Alzheimer’s disease Word Learning sub-test; AFT, Animal Fluency Test; DSST, Digit Symbol Substitution Test; Z-score was calculated by averaging the z-scores of CERAD, AFT, and DSST.

Table 3 showed that engagement in sufficient moderate to high-intensity recreation activity and walking/bicycling were associated with lower depression score as revealed by weighted multivariable linear regression. Compared with insufficient moderate to high-intensity recreation activity and walking/bicycling, sufficient moderate to high-intensity recreation activity (β: −1.309; 95% CI: −1.653 to −0.966) and walking/bicycling (β:−1.183; 95% CI: −1.683 to −0.682) demonstrated a statistically significant inverse association with depression score. The findings remained consistent in the fully adjusted model.

**TABLE 3:** Associations between physical activity and depression score [β (95% CI)].

Physical activity	Weighted number (%)	Crude model^£^	*P*-value	Adjusted model^§^	*P*-value
**Moderate to high-intensity recreation activity (min/week)**					
< 150	34,575,950 (69.8)	Ref		Ref	
≥ 150	14,963,694 (30.2)	−1.309 (−1.653, −0.966)	< 0.001	−0.861 (−1.233, −0.49)	< 0.001
**Moderate to high-intensity work activity (min/week)**					
< 150	37,079,450 (74.8)	Ref		Ref	
≥ 150	12,460,195 (25.2)	−0.364 (−0.851, 0.123)	0.138	−0.091 (−0.567, 0.385)	0.687
**Walking/bicycling (min/week)**					
< 150	43,574,216 (88.0)	Ref		Ref	
≥ 150	5,965,429 (12.0)	−1.183 (−1.683, −0.682)	< 0.001	−1.039 (−1.593, −0.485)	0.001

^£^ Crude model adjusted for no confounding variables.

^§^ Adjusted model adjusted for age, gender, ethnicity, marital, education, BMI, smoking, total cholesterol/high density lipoprotein cholesterol, hypertension, diabetes, cardiovascular disease, lipid-lowering drugs, antihypertensive drugs, hypoglycemic agents, antiplatelet drugs.

**FIGURE 3:**
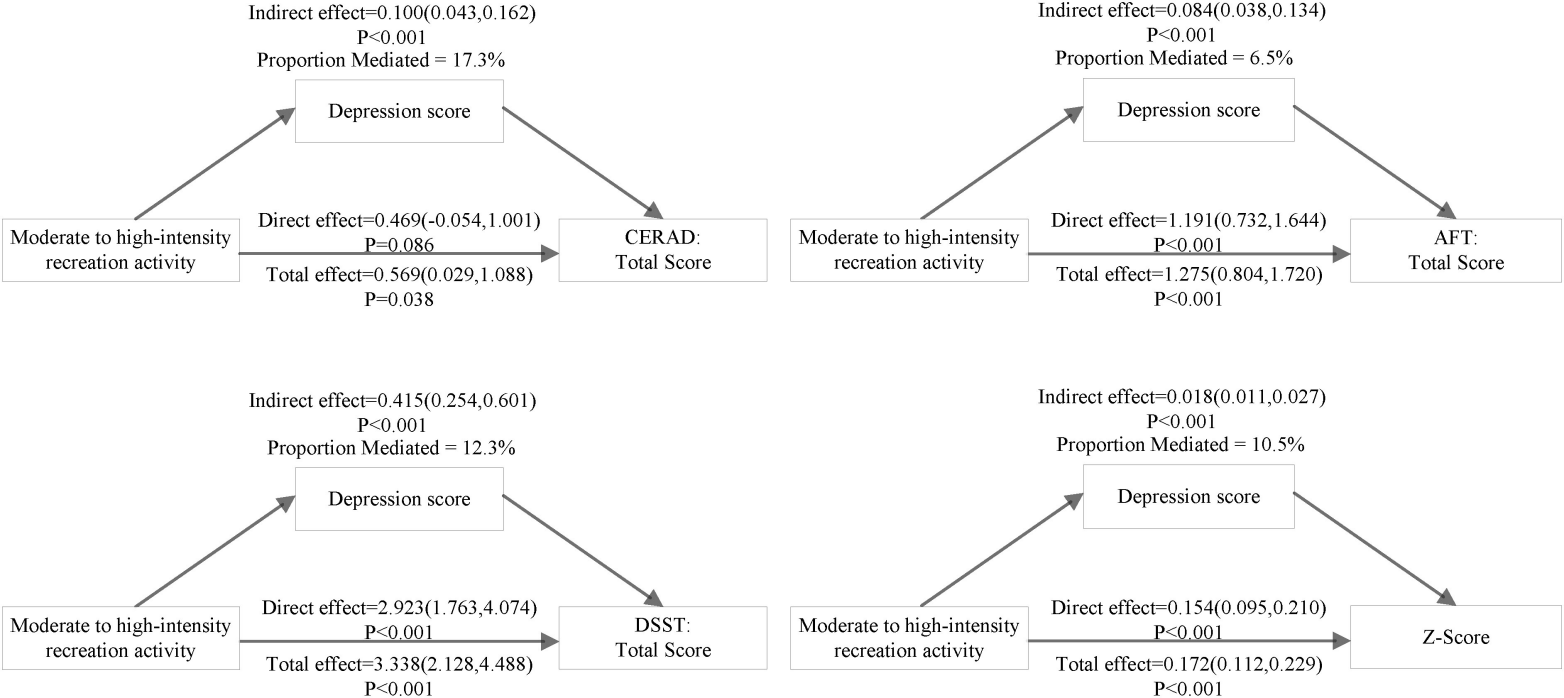
Path diagram of mediation model present the effect of moderate to high-intensity recreation activity on Consortium to Establish a Registry for Alzheimer’s Disease (CERAD), Animal Fluency Test (AFT), Digit Symbol Substitution Test (DSST), and composite z-score mediated by depression score in full participants. Model adjusted for age, gender, ethnicity, marital, education, BMI, smoking, total cholesterol/high density lipoprotein cholesterol, hypertension, diabetes, cardiovascular disease, lipid-lowering drugs, antihypertensive drugs, hypoglycemic agents, antiplatelet drugs.

Table 4 presented the results of mediation analysis. Adjusting for covariates, it was found that depression mediated the association between moderate to high-intensity recreation activity and cognitive function scores (CERAD, AFT, DSST, and z-score). The total effect of moderate to high-intensity recreation activity on z-score was 0.172 (95% CI: 0.112 to 0.229), the indirect effect of moderate to high-intensity recreation activity through depression was 0.018 (95% CI: 0.011 to 0.027), accounting for a mediated proportion of 10.5%. Likewise, the mediation proportions of depression on CERAD, AFT, and DSST were 17.3%, 6.5% and 12.3%, respectively. And the corresponding visual pattern diagram is shown in Figure 3.

**TABLE 4:** The mediating effect of depression score on the association between physical activity and cognitive function in full participants (*n* = 2,681).

Physical activity	CERAD: total score	AFT: total score	DSST: total score	Z-score
**Crude model^£^**				
** Moderate to high-intensity recreation activity**				
Indirect effect, β (95% CI)	0.145 (0.063, 0.240)	0.172 (0.103, 0.253)	0.871 (0.617, 1.646)	0.035 (0.024, 0.048)
*P*-value	< 0.001	< 0.001	< 0.001	< 0.001
Direct effect, β (95% CI)	1.034 (0.461, 1.582)	1.765 (1.263, 2.239)	6.200 (4.727, 7.627)	0.281 (0.211, 0.349)
*P*-value	< 0.001	< 0.001	< 0.001	< 0.001
Total effect, β (95% CI)	1.179 (0.621, 1.711)	1.937 (1.452, 2.401)	7.071 (5.630, 8.472)	0.316 (0.248, 0.382)
*P*-value	< 0.001	< 0.001	< 0.001	< 0.001
Proportion mediated (%)	12.1	8.9	12.3	11.0
**Adjusted model^§^**				
** Moderate to high-intensity recreation activity**				
Indirect effect, β (95% CI)	0.100 (0.043, 0.162)	0.084 (0.038, 0.134)	0.415 (0.254, 0.601)	0.018 (0.011, 0.027)
*P*-value	< 0.001	< 0.001	< 0.001	< 0.001
Direct effect, β (95% CI)	0.469 (−0.054, 1.001)	1.191 (0.732, 1.644)	2.923 (1.763, 4.074)	0.154 (0.095, 0.210)
*P*-value	0.086	< 0.001	< 0.001	< 0.001
Total effect, β (95% CI)	0.569 (0.029, 1.088)	1.275 (0.804, 1.720)	3.338 (2.128, 4.488)	0.172 (0.112, 0.229)
*P*-value	0.038	< 0.001	< 0.001	< 0.001
Proportion mediated (%)	17.3	6.5	12.3	10.5

^£^ Crude model adjusted for no confounding variables.

^§^ Adjusted for age, gender, ethnicity, marital, education, BMI, smoking, total cholesterol/high density lipoprotein cholesterol, hypertension, diabetes, cardiovascular disease, lipid-lowering drugs, antihypertensive drugs, hypoglycemic agents, antiplatelet drugs. CERAD, Consortium to Establish a Registry for Alzheimer’s disease; Word Learning sub-test; AFT, Animal Fluency Test; DSST, Digit Symbol Substitution Test; Z-score was calculated by averaging the z-scores of CERAD, AFT, and DSST.

**FIGURE 4:**
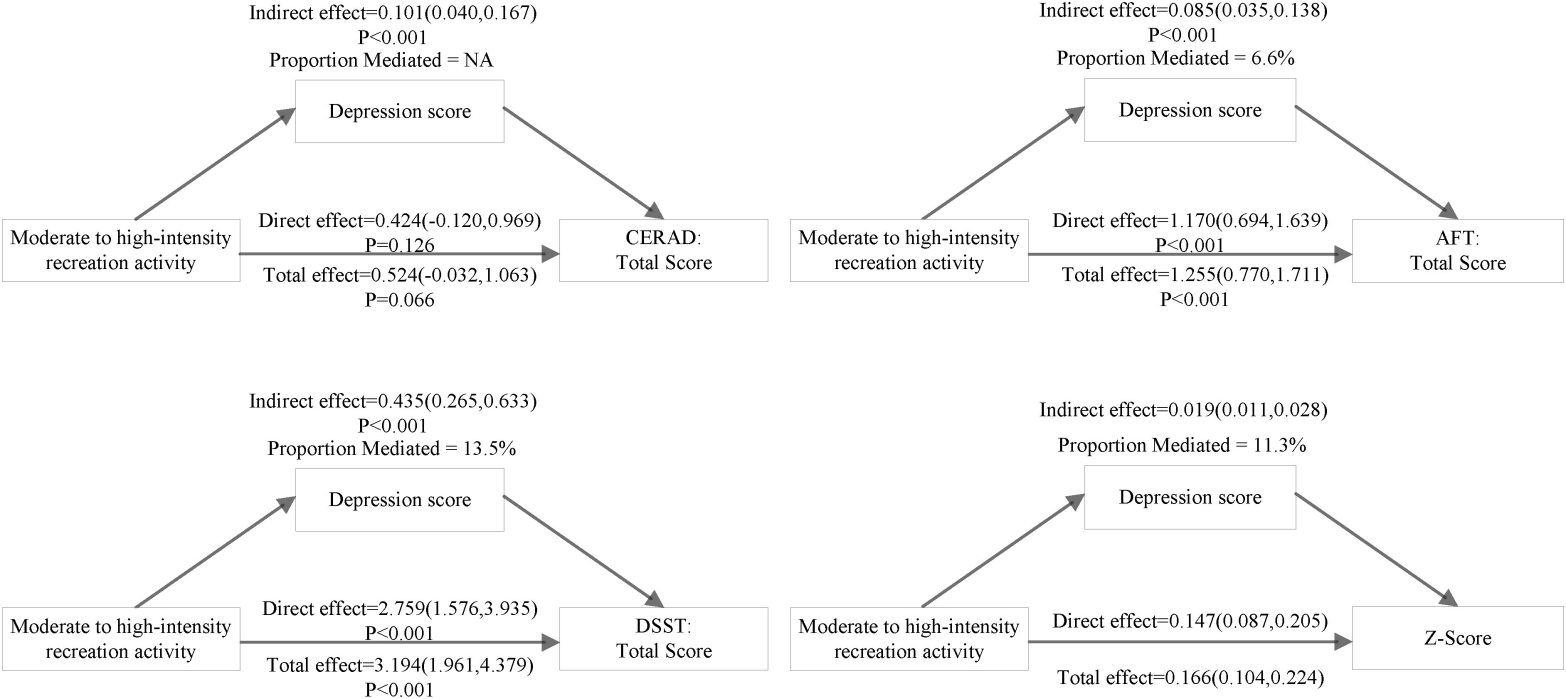
Path diagram of mediation model present the effect of moderate to high-intensity recreation activity on Consortium to Establish a Registry for Alzheimer’s Disease (CERAD), Animal Fluency Test (AFT), Digit Symbol Substitution Test (DSST), and composite z-score mediated by depression score in non-stroke participants. Model adjusted for age, gender, ethnicity, marital, education, BMI, smoking, total cholesterol/high density lipoprotein cholesterol, hypertension, diabetes, cardiovascular disease, lipid-lowering drugs, antihypertensive drugs, hypoglycemic agents, antiplatelet drugs.

After further excluding participants with a history of stroke, the sensitivity analysis of mediating role was presented in Table 5. In sensitivity analysis, after adjusted for all confounders, the mediating effect of depression score remained significant between moderate to high-intensity recreation activity and three cognitive functions measures (AFT, DSST, and z-score). The mediation proportions of depression on z-score, AFT, and DSST were 11.3%, 6.6%, and 13.5%, respectively. And the corresponding visual pattern diagram was shown in Figure 4.

**TABLE 5:** Sensitivity analysis for the mediating effect of depression score on the association between physical activity and cognitive function in non-stroke participants (*n* = 2,503).

Physical activity	CERAD: total score	AFT: total score	DSST: total score	Z-score
**Crude model^£^**				
** Moderate to high-intensity recreation activity**				
Indirect effect, β (95% CI)	0.133 (0.046, 0.231)	0.167 (0.096, 0.251)	0.858 (0.600, 1.160)	0.034 (0.022, 0.047)
*P*-value	0.002	< 0.001	< 0.001	< 0.001
Direct effect, β (95% CI)	0.996 (0.406, 1.561)	1.709 (1.192, 2.198)	6.060 (4.544, 7.529)	0.273 (0.201, 0.343)
*P*-value	< 0.001	< 0.001	< 0.001	< 0.001
Total effect, β (95% CI)	1.129 (0.554, 1.677)	1.875 (1.376, 2.354)	6.918 (5.437, 8.358)	0.307 (0.237, 0.375)
*P*-value	< 0.001	< 0.001	< 0.001	< 0.001
Proportion mediated (%)	11.6	8.8	12.4	10.9
**Adjusted model^§^**				
** Moderate to high-intensity recreation activity**				
Indirect effect, β (95% CI)	0.101 (0.040, 0.167)	0.085 (0.035, 0.138)	0.435 (0.265, 0.633)	0.019 (0.011, 0.028)
*P*-value	< 0.001	< 0.001	< 0.001	< 0.001
Direct effect, β (95% CI)	0.424 (−0.120, 0.969)	1.170 (0.694, 1.639)	2.759 (1.576, 3.935)	0.147 (0.087, 0.205)
*P*-value	0.126	< 0.001	< 0.001	< 0.001
Total effect, β (95% CI)	0.524 (−0.032, 1.063)	1.255 (0.770, 1.711)	3.194 (1.961, 4.379)	0.166 (0.104, 0.224)
*P*-value	0.066	< 0.001	< 0.001	< 0.001
Proportion mediated (%)		6.6	13.5	11.3

^£^ Crude model adjusted for no confounding variables.

^§^ Adjusted for age, gender, ethnicity, marital, education, BMI, smoking, total cholesterol/high density lipoprotein cholesterol, hypertension, diabetes, cardiovascular disease, lipid-lowering drugs, antihypertensive drugs, hypoglycemic agents, antiplatelet drugs. CERAD, Consortium to Establish a Registry for Alzheimer’s disease; Word Learning sub-test; AFT, Animal Fluency Test; DSST, Digit Symbol Substitution Test; Z-score was calculated by averaging the z-scores of CERAD, AFT, and DSST.

## Discussion

This cross-sectional study utilized data from NHANES cycles 2011–2012 and 2013–2014 to examine the potential role of depression in the relationship between PA and cognitive function among older adults. Our findings revealed that higher levels of moderate- to high-intensity recreation activity are associated with improved cognitive function, particularly in relation to performance on the CERAD, AFT, DSST, and z-score. Furthermore, the study demonstrated that depression partially mediates the effects of moderate- to high-intensity recreation activity on cognitive function. These results provide novel insights into how PA, especially moderate- to high-intensity recreation activity may contribute to cognitive enhancement, highlighting the potential mediating role of depression in this association.

The results regarding the relationship between moderate- to high-intensity recreation activity and cognition are generally consistent with those of previous studies (Lautenschlager et al., 2008; Lim et al., 2020; Jeong et al., 2021). Older Koreans who met the recommended levels of moderate- to high-intensity physical activity exhibited approximately a 2-fold greater improvement in cognitive function compared to those who did not meet the recommendations (Lim et al., 2020). Lautenschlager et al. (2008) demonstrated that 150 min per week of moderate-intensity physical activity can delay the age-related decline in cognitive function. A randomized controlled trial showed that a 12 weeks moderate- to high-intensity physical activity program significantly improved cognitive function in individuals with MCI compared to control groups (Jeong et al., 2021). This intervention has been recognized as an effective strategy for dementia prevention through enhancements in overall and frontal lobe cognitive function (Jeong et al., 2021). Furthermore, an earlier study indicated that moderate- to high-intensity physical activity, as opposed to low- intensity, increases the volume of the anterior hippocampus, thereby improving spatial memory (Erickson et al., 2011). At the molecular level, moderate- to high-intensity physical activity may enhance cognition by increasing brain-derived neurotropic factor (BDNF), neurogenesis, and synaptic plasticity (Erickson et al., 2011; Ratey and Loehr, 2011). However, in this study, no significant association was observed between moderate- to high-intensity work activity, walking/bicycling, and cognitive function. Differences in self-motivation may account for this discrepancy (Huang et al., 2021). A general population study in Copenhagen showed that occupational PA and leisure time PA have opposing effects on the risk of major adverse cardiovascular events and all-cause mortality, supporting the concept of the physical activity paradox (Holtermann et al., 2021).

This study found that the effect of moderate- to high-intensity recreation activity on cognitive function was partially mediated by depression. Australian men who engaged in at least 2.5 h per week of moderate- to high-intensity physical activity were 40% less likely to report moderate to high levels of depression over the past 2 weeks compared to those with lower levels of activity (Currier et al., 2020). In a cross-national sample of older adults in the United States, objectively measured moderate- to high-intensity physical activity was inversely associated with depressive symptoms (Loprinzi, 2013). In the context of depression, PA has been shown to reduce symptom severity, lower the risk of onset, and decrease the likelihood of relapse (Currier et al., 2020), suggesting its potential as an alternative to antidepressants in primary care settings (López-Torres Hidalgo, 2019). Possible mechanisms by which PA improves depression include distraction from negative thoughts and increased social interaction (López-Torres Hidalgo, 2019). PA may induce changes in the functional activity of mood-related monoamines, which could influence cognitive changes (Craft and Perna, 2004). Monoamine-mediated signaling pathways may contribute to the regulation of the BDNF gene and enhance BDNF expression in the hippocampus (Cotman and Berchtold, 2002). Therefore, it has been suggested that depression has a causal relationship on cognitive function (Dotson et al., 2008; McDermott and Ebmeier, 2009). A growing body of research has demonstrated that depression is strongly associated with poorer cognitive performance (Dotson et al., 2008; McDermott and Ebmeier, 2009; Wu et al., 2021). Meta-analytic studies examining the relationship between cognitive function and depression severity have identified significant associations between cognitive domains such as episodic memory, executive function, and processing speed and the severity of depressive symptoms (McDermott and Ebmeier, 2009). Longitudinal analyses have further shown that depression is linked to cognitive decline, particularly with advancing age (Dotson et al., 2008; Wu et al., 2021). Overall, regular PA may partially improve cognitive function through its beneficial effects on depression. Depression played a mediation role between PA and cognition function. Therefore, targeted recreational active PA interventions may be beneficial for maintaining better cognitive function, thereby delaying the occurrence of dementia.

A strength of this study lies in the use of a nationally representative dataset of United States older adults, which ensures a more objective and unbiased assessment of this population and enhances the reliability of identifying associations among the studied factors. Furthermore, this study is the first to examine depression as a mediating factor in the relationship between PA and cognitive function, addressing an important research gap in the field of cognitive aging. Our findings indicate that the average depression score among community-dwelling older adults is relatively low. Nevertheless, depression still partially mediates the effect of PA on cognitive function. This result may offer valuable insights: early screening and intervention for depression in older adults could play a significant role in preserving cognitive health. Despite these contributions, several limitations should be acknowledged. First, this study is a cross-sectional study, which limits our exploration of the causal relationship between the studied factors. Second, the reliance on self-reported PA data may introduce recall bias, which could affect measurement accuracy and lead to reporting bias. Moreover, our results show that depression only partially accounts for the association between PA and cognitive function. Further research is needed to explore additional potential mediating or moderating factors that may influence this relationship. These limitations should be considered when interpreting the findings and highlight important directions for future studies.

## Conclusion

Our study highlights that increased engagement in moderate- to high-intensity recreation activity is associated with improved cognitive performance in older adults. Notably, depression is identified as playing a certain mediating role in this relationship. To help maintain better cognitive function in aging populations, our findings suggest the importance of actively participating in recreational physical activity while simultaneously addressing and managing depressive symptoms. These insights contribute to a broader understanding of lifestyle factors that influence cognitive health in older adults.

## Data Availability

Publicly available datasets were analyzed in this study. This data can be found here: NHANES database (https://www.cdc.gov/nchs/nhanes/index.htm).
